# Psychometric Properties of a Russian Version of the Cognitive Flexibility Inventory (CFI-R)

**DOI:** 10.3389/fpsyg.2018.00845

**Published:** 2018-05-29

**Authors:** Sergey S. Kurginyan, Ekaterina Y. Osavolyuk

**Affiliations:** ^1^School of Psychology, Scientific-Educational Laboratory of Ability Psychology, National Research University Higher School of Economics, Moscow, Russia; ^2^Doctoral School of Psychology, National Research University Higher School of Economics, Moscow, Russia

**Keywords:** cognitive flexibility, Cognitive Flexibility Inventory, coping, depression, rigidity

## Abstract

The Cognitive Flexibility Inventory (CFI) is a brief self-report measure of the type of cognitive flexibility (CF) necessary to successfully challenge and restructure maladaptive beliefs with more balanced and adaptive thinking; it is particularly popular for use with English speakers. The CFI has recently been translated into five languages (Chinese, Japanese, Iranian, Turkish, and Russian), although estimates of reliability and validity of these translated versions are scarce. This study reports on the factor structure, internal consistency, reliability, and construct validity of the CFI. We adopted the CFI for a Russian-speaking population, using student sample of 445 first and second-year undergraduates (*M* = 18.59 years, *SD* = 1.19) and found that a two-factor model fitted the data well. However, the structure of the CFI was revised because of some modifications, which were made to the original English to match the Russian equivalents of items originally developed to assess the definite aspect of cognitive flexibility. The CFI-R showed good internal consistency and suitable 7-week test–retest reliability. The construct validity of the Russian version of the CFI was studied by computing correlations with other related measures of CF (Attributional Style Questionnaire), depressive symptoms (Beck Depression Inventory), coping (Ways of Coping (Revised), and rigidity (Tomsk Rigidity Questionnaire). Furthermore, to assess whether the construct validity were affected by psychopathology we examined results for non-clinical and clinical samples, using “known-groups” method. The clinical sample reported lower CF than did the non-clinical sample on the CFI-R’s total score and its subscales’ scores. Findings in the present study suggest that the psychometric properties of the Russian CFI are comparable to the English original, making it appropriate to research assessment of the type of CF in Russian speaking population.

## Introduction

According to Dennis and Vander Wal (2010) the ability to switch cognitive sets to adapt to changing environmental stimuli is a core component for most operational definitions of cognitive flexibility (CF). It manifests itself in a broad spectrum of behaviors that are considered to enable individuals to think adaptively rather than maladaptively when encountering stressful life events. Despite the fact that the CF has been well-researched, and that some of the researchers adopt differing approaches to studying the related phenomena (Ionescu, 2012, 2017), it seems that this construct has not easy to define and assess. Historically, CF has been defined as a cognitive mechanism or ability of executive functioning (Rende, 2000) and has been assessed using performance-based measures such as the Trail Making Test Part B (TMT-B; Reitan and Wolfson, 1993), Stroop Color and Word Test (Golden, 1978), Wisconsin Card Sorting Test (WCST; Heaton et al., 1993) and self-report measures such as the Alternate Uses Test (Wilson et al., 1975), Attributional Style Questionnaire (ASQ; Peterson et al., 1982). However, little is known about the potential value of existing measures within a treatment context.

The self-report measures are attractive for psychological treatment because of “they are brief, can be completed independently, and can be quickly scored and interpreted” (Johnco et al., 2014, p. 1382). The self-report format are likely to assess the CF required to restructure maladaptive thoughts, as it is more state like and reactive to affective states (Dennis and Vander Wal, 2010). At present, two self-report measures are the most widely used instruments of CF within treatment practice: the Cognitive Flexibility Scale (CFS; Martin and Rubin, 1995) and Cognitive Flexibility Inventory (CFI; Dennis and Vander Wal, 2010), although neither has been tested on non-clinical or clinical samples in the Russian context. As the CFI was specifically designed to measure aspects of CF that enable individuals to think adaptively rather than maladaptively when encountering stressful life events (Dennis and Vander Wal, 2010), we chose to validate it in the Russian-speaking population.

The CFI is a brief 20-item, two-subscale self-report measure, designed to assess aspects of CF that enable individuals to think adaptively rather than maladaptively when encountering stressful life events. It provides important information for professionals by monitoring the levels of CF evidenced by individuals engaged in cognitive behavioral thought challenging interventions. The CFI was tested on a student sample consisted of 196 undergraduates in their junior or senior years of college (mean age: 20.20 ± 1.05 years, 75% female, 81% Caucasian). The principal axes extraction method yielded a two-factor structure with 20 items loading on two factors corresponding to the following subscales: Alternatives and Control, all showing good to excellent internal consistency (αs ranging from 0.84 to 0.91). The Alternatives subscale is composed of 13 items, all of which measure the ability to perceive multiple alternative explanations for life occurrences and human behavior and the ability to generate multiple alternative solutions to difficult situations; the Control subscale is composed of 7 items, most of which were originally developed to measure the tendency to perceive difficult situations as controllable. The measure demonstrated high 7-week test–retest reliability for the CFI total score and its subscales (*r* = 0.75–0.81; *p* < 0.001), as well as convergent construct validity of the CFI and its two subscales via their associations with other measures of CF (CFS; Martin and Rubin, 1995; ASQ; Peterson et al., 1982), depressive symptomatology (BDI-II; Beck et al., 1996), and coping (WCCL-R; Folkman and Lazarus, 1985).

A number of subsequent studies have utilized this measure of CF in non-clinical and clinical situations and reported its good psychometric features across countries and samples. Although the CFI has been largely applied internationally, studies examining its structural validity have shown different results. The two-factor structure and internal consistency have been confirmed for the CFI in Turkish (Gülüm and Daǧ, 2012), Japanese (Tokuyoshi and Iwsaki, 2012), Chinese (Wang et al., 2016), and Russian preliminary version (Kurginyan and Osavolyuk, 2018). Shareh et al. (2014), applying an Iranian version of the instrument to university students failed to replicate the factor structure reported by the CFI’s developers. These researchers suggested a three-factor solution and considered it optimal for the Iranian version of the CFI. The results of EFA by principle component analysis method with Varimax rotation indicated greater variance (56.02%) and supported the modified scale with 20 item loadings on three correlated factors: Control (8 items), Alternatives (10 items), and Alternatives for Human Behaviors (2 items). Factor scores were positively correlated with resilience and coping strategies considered to be adaptive and negatively correlated with depression and coping strategies considered to be maladaptive, except the non-significant correlations of Alternatives for Human Behaviors subscale (Shareh et al., 2014). Moreover, studies confirming a structural validity of the CFI reported some corrections of items loading to the original two-factor model. In particular, Gülüm and Daǧ (2012) replicated the factor structure of the CFI on a non-clinical sample in the Turkish context indicated that item 19 had better factor loading on Control subscale (0.43; α = 0.85). In our preliminary study of the initial psychometrics of the Russian CFI tested on a non-clinical sample of university students (Kurginyan and Osavolyuk, 2018) we reproduced a two-factor solution that was somewhat different from the two-factor solution of the original developers of the English version. Differences were made to the original CFI by transferring items 1, 2 and 15, originally had acceptable factor loadings on the Alternatives subscale, to the Control subscale, and items 14 and 19, originally had acceptable factor loadings on the Control subscale, to the Alternatives subscale. A newly specified structure of the CFI fitted the data well χ^2^(*N* = 298) = 439.42, *df* = 169, *p* = 0.001, GFI = 0.88, CFI = 0.87, SRMR = 0.07, RMSEA = 0.07, 90% CI [0.07, 0.08]. In summary, considering the construct validity and reliability of the CFI across countries and samples, these studies demonstrated inconsistent findings of the factor structure. To date, in China with a college student sample, Wang et al. (2016) have indicated good fit (χ~2/*df* = 3.52, CFI = 0.90, NNFI = 0.89, SRMR = 0.06, RMSEA = 0.06) providing support for the replicability of the original CFI.

As there is currently no instrument in Russian that can provide a valid and reliable measure of CF within a research investigation or therapeutic intervention, the present study aims to explore the replicability of the factor structure and the psychometric properties of the CFI on university students, and to develop its Russian version (CFI-R). Our analysis focuses on the CFI-R factor structure, reliability, and construct validity. We predicted to indicate the negative associations of CF aspects measured by CFI-R with depressive symptomology, coping strategies considered to be maladaptive and personality rigidity.

## Material and Methods

### Participants

The present study included non-clinical and clinical samples. The non-clinical sample consisted of 445 first and second-year undergraduate students of the National Research University “Higher School of Economics” (357 females, 88 males), ranging in age from 16 to 25 years (*M* = 18.59, *SD* = 1.19). The majority of students were Russian (96%), and in social (34%) and human sciences (66%) according to the field of study. The clinical sample consisted of 35 outpatients (32 females and 3 males), ranging in age from 21 to 82 years (*M* = 49.66, *SD* = 17.68). According to the *Diagnostic and Statistical Manual of Mental Disorders* (5th Edn; DSM-5; American Psychiatric Association, 2013), depressive disorders were predominantly occurring diagnoses (63% of the total), followed by schizophrenia spectrum and other psychotic disorders (26%), bipolar and related disorders (3%), and anxiety disorder (8%).

### Procedure

Students were invited to participate in a questionnaire study. Those who agreed, completed the CFI (Dennis and Vander Wal, 2010), ASQ (Peterson et al., 1982), Beck Depression Inventory (BDI; Beck et al., 1961), Ways of Coping (Revised) (WC-R; Folkman and Lazarus, 1985), and Tomsk Rigidity Questionnaire (TRQ; Zalevsky, 2007). All instruments were administrated in Russian. The questionnaire session required approximately 45 min to complete. The students earned course extra credits for their participation. All 445 students who participated in the first group questionnaire session (testing) were invited to return for a second group questionnaire session (re-testing) 7 weeks later. They earned additional course extra credits for their participation in this second group questionnaire session. To recruit the clinical sample, we contacted four clinical mental hospitals and asked if they were willing to participate in a questionnaire study. Psychiatrists from one hospital that agreed presented an informational letter to their patients. We explained our goals in the letter and asked them to participate in the study by completing the questionnaire. Those who agreed filled out the Russian translation of the CFI in hospital and returned it to us via the psychiatrists. Data were collected between October 2015 and May 2016. The Higher School of Economics (HSE) Committee on Interuniversity Surveys and Ethical Assessment of Empirical Research approved the study, which was conducted in accordance with the standards of the Code of Ethics of the Russian Psychological Society.

### Measures

#### Cognitive Flexibility Inventory (CFI)

This 20-item self-report measure is designed to assess the levels of CF evidenced by individuals engaged in cognitive behavioral thought challenging interventions (Dennis and Vander Wal, 2010). The CFI items consist of statements dealing with beliefs and feelings about behavior for which individuals could indicate their agreement or disagreement. The CFI comprises two subscales, the Alternatives and Control subscale that measure three aspects of cognitive flexibility: (a) the tendency to perceive difficult situations as controllable; (b) the ability to perceive multiple alternative explanations for life occurrences and human behavior; and (c) the ability to generate multiple alternative solutions to difficult situations. Each item is scored on a seven-point Likert scale, ranging from strongly disagree (1) to strongly agree (7). The scoring procedures specified for the CFI require reverse scoring of select items and then summing the numerical response values to obtain a total score. Higher scores on both scales are indicative of greater cognitive adaptability associated with greater CF when encountering stressful situations; lower scores are indicative of greater cognitive rigidity associated with less cognitive adaptability when encountering stressful situations (Dennis and Vander Wal, 2010). The Russian translation of the CFI was made by front-and-back translation procedure and has provided the initial psychometrics in the Russian population: internal consistency for CFI total score, Alternatives and Control subscales (α = 0.86, 0.77, 0.81, respectively) and 7-week test–retest reliability (*r* = 0.66, 0.52, 0.71, respectively; *p* < 0.01) (Kurginyan and Osavolyuk, 2018).

#### Beck Depression Inventory (BDI; Beck et al., 1961)

The BDI is a 21-item self-report rating inventory that measures characteristic attitudes and symptoms of depression. Participants are instructed to choose one statement from the four listed beneath each item that best describes the way they have been feeling during the past week including that day. Each item is scored from 0 to 3, with higher scores indicating more serious depressive symptoms. The scores from 0 to 9 are regarded as minimal depressive symptoms, 10–18 as mild depression, 19–29 as moderate depression, and 30–63 as severe depression. The reliability and validity of the Russian translation of the BDI (α = 0.86) and its “cognitive-affective” (α = 0.79) and somatic-performance subscales (α = 0.79) have been confirmed by Tarabrina (2001) when used with a sample of university students.

#### Attributional Style Questionnaire (ASQ; Peterson et al., 1982)

The ASQ is a 48-item self-report measure of explanatory style for good and bad events. It presents 12 hypothetical events (six positive and six negative). The respondents are asked to write down one major cause of each event and then rate the cause along a seven-point Likert scale (1–7) continuum for each of the three causal dimensions: internal versus external, stable versus unstable, and global versus specific causes. The original authors (Peterson et al., 1982) reported reliability (0.67) and validity (α = 0.72–0.75) of the ASQ. The psychometric properties of the Russian translation are acceptable and comparable to those of the original ASQ (Gordeeva et al., 2008). In the present research more extreme scores on the Likert scale were considered indicative of less CF on the ASQ (Teasdale et al., 2001; Dennis and Vander Wal, 2010).

#### Ways of Coping (Revised) (WC-R; Folkman and Lazarus, 1985)

The WC-R is a self-report measure of coping derived from Lazarus’ transactional model of stress. It contains 50 items representing thoughts and actions which can be used to deal with the demands of a stressful encounter. Responses to the WC-R are scored on a four-point Likert scale: *does not apply and/or not used* (0); used somewhat (1); used quite a bit (2); used a great deal (3). Folkman and Lazarus (1985) reported that the WC-R has an eight-factor structure: Confrontive Coping (CC; α = 0.70); Distancing (D; α = 0.61); Self-controlling (Sc; α = 0.70); Seeking Social Support (SSS; α = 0.76); Accepting Responsibility (AR; α = 0.66); Escape-Avoidance (SB; α = 0.72); Planful Problem-Solving (PPR; α = 0.68); and Positive Reappraisal (PR; α = 0.68). Recently, Vasserman et al. (2009) evaluated the psychometric properties of the Russian translation positively.

#### Tomsk Rigidity Questionnaire (TRQ; Zalevsky, 2007)

This is a 150-item eight-subscale self-report questionnaire, to which responses are made on a four-point Likert scale: *definitely disagree* (0), *probably disagree* (1), *probably agree* (3), *definitely agree* (4). The TRQ was developed to capture mental rigidity that a person experiences related to situations that challenge to change individual elements of a behavior program depending on the lifestyle, stereotypes, relations, attitudes, habits, skills, life and activity rate and rhythm, means of goal attainment, etc. The TRQ (α = 0.92) comprises six main subscales, which measure the different aspects of mental rigidity, and two control scales, which measure whether the respondent has indicated the agreement to the Tomsk Rigidity Questionnaire’s statements from own experience or from some assumptions (Reality scale; 17 items; α = 0.70), and whether the respondent has answered truthfully in other parts of the questionnaire (Lie scale^1^; 9 items; α = 0.59). The Rigidity Symptom Complex subscale (63 items; α = 0.87) reflects a person’s predisposition to a wide range of forms of stabile behavior such as perseveration, obsession, stereotypy, obstinacy, pedantry, and rigidity proper. The latter aspect of this subscale is reflected in the Actual Rigidity subscale (17 items; α = 0.76), which measures a person’s inability to change their opinion, relation, attitude, motives, manner of experiencing, and etc. The Sensitive Rigidity subscale (18 items; α = 0.79) reflects a person’s emotional reaction to anything unfamiliar in situations regarding any chances (similarly to neophobia). The Attitudinal Rigidity subscale (17 items; α = 0.58) reflects a personality level of mental rigidity manifestation expressed in a person’s set, relation or attitude to acceptance/rejection of anything new, need to change the self-concept, level of aspiration, habit complex, and etc. The Rigidity as a State subscale (6 items; α = 0.79) reflects a person’s tendency to rigid (fixed) behavior being in a state of fear, stress (distress), impaired mood, fatigue, or sickliness. The Premorbid Rigidity (20 items; α = 0.67) subscale reflects whether a person has experienced difficulties in situations regarding any chances being in teen-age and preadult age. Respondents retrospectively assess how they have behaved, undergone and overcome any difficulties at school age (for patients it is a premorbid period). In the normal differential estimate of mental rigidity there are four levels of its display: “low,” “moderate,” “high,” and “very high.” The original author (Zalevsky, 2007) has shown that TRQ is valid to measure Rigidity and reliable when used with a university student sample. We assessed the internal consistency of the TRQ and indicated Cronbach’s alpha coefficient for its scales in present study.

### Data Analysis

Descriptive statistics, exploratory factor analysis (EFA), internal consistency, and intercorrelations of scales were calculated using the statistical package IBM SPSS Statistics (Version 22). Cronbach’s alpha was used to examine the internal consistency of the Russian version of the Cognitive Flexibility Inventory (CFI-R), Pearson’s correlation coefficient and intra-class correlation coefficients (ICC) were used to establish the 7-week test–retest reliability. The CFI-R’s construct validity and its two subscales were assessed through calculating correlations with related measures comprising the BDI, ASQ, WC-R, and TRQ. An EFA was performed to examine the factor structure of the CFI using a principal axes factor analysis. A Promax rotation with Kaiser normalization was utilized to test a hypothesis that two factors of the CFI are correlated, as it was anticipated by original developers of English version (Dennis and Vander Wal, 2010). Student’s *t*-statistic was used to assess the significance of difference in the CFI-R’s total scores and its subscales scores of the two samples (non-clinical and clinical samples). All other analyses were carried out by applying confirmatory factor analysis (CFA) using EQS 6.2 for Windows (Bentler, 2006). Since the data was not normally distributed, the maximum likelihood procedure with non-normally robust standard errors was used to assess the model parameters. We applied multiple fit indices, including Satorra and Bentler (1988) scaled χ^2^ statistic, the comparative fit index (CFI), goodness-of-fit index (GFI), the standardized root mean square residual (SRMR), the root-mean-square error of approximation (RMSEA) and confidence interval (CI), to evaluate model fit (McDonald and Ho, 2002; Kline, 2011).

## Results

### Factorial Validity

To assess the structure of the CFI, we first ran an EFA. The Kaiser-Meyer-Olkin (KMO) measure of sampling adequacy (Kaiser, 1970) and Bartlett’s test of sphericity demonstrated that item bivariate correlations were adequate for factorability (0.89 and 3055.78, respectively, at *p* < 0.0001). Based on the Scree plot and Kaiser’s coefficient alpha of generalizability (Kline and Barrett, 1983), a two-factor solution was considered best for our data set. The minimal item loadings on a factor were set at >0.32. This two-factor solution accounted for 37.14% of the total variance. The factor loadings are shown in **Table 1**. It can be seen from the table that the first factor was composed of 12 items, 10 of which were originally included in the Alternatives subscale and two items were originally included in the Control subscale. Item 14: “I often look at a situation from different viewpoints” and item 19: “I can think of more than one way to resolve a difficult situation I’m confronted with” had acceptable factor loadings on the Alternatives subscale but, if deleted, the internal consistency reliability would reduce. The Russian equivalents of these two items were further examined for how well they matched the construct that measured aspects *b* and *c* of CF (Dennis and Vander Wal, 2010). Giving their factor loadings and closeness in meaning, they retained in the Alternatives subscale. The second factor was composed of eight items, five of which were originally included in the Control subscale and three items were originally included in the Alternatives subscale. Item 1: “I am good at ‘sizing up’ situations,” item 2: “I have a hard time making decisions when faced with difficult situations,” and item 15: “I am capable of overcoming the difficulties in life that I face” had acceptable factor loadings on the Control subscale. After reconsidering their meaning in Russian and examining for how well they matched construct that measured aspect *a* (Dennis and Vander Wal, 2010), they were ascribed to the Control subscale.

**Table 1:** Factor loadings for the CFI retained after exploratory factor analysis.

CFI items		Factor 1	Factor 2
**Item 13**	**When in difficult situations, I consider multiple options before deciding how to behave.**	**0.79**	
Item 14	I often look at a situation from different viewpoints.	0.79	
**Item 5**	**I like to look at difficult situations from many different angles.**	**0.64**	
**Item 20**	**I consider multiple options before responding to difficult situations.**	**0.64**	
**Item 3**	**I consider multiple options before making a decision.**	**0.59**	
**Item 12**	**It is important to look at difficult situations from many angles.**	**0.55**	
Item 19	I can think of more than one way to resolve a difficult situation I’m confronted with.	0.50	
**Item 6**	**I seek additional information not immediately available before attributing causes to behavior.**	**0.48**	
**Item 16**	**I consider all the available facts and information when attributing causes to behavior.**	**0.46**	
**Item 18**	**When I encounter difficult situations, I stop and try to think of several ways to resolve it.**	**0.44**	
**Item 8**	**I try to think about things from another person’s point of view.**	**0.35**	
**Item 10**	**I am good at putting myself in others’ shoes.**	**0.32**	
Item 11	When I encounter difficult situations, I just don’t know what to do.		0.83
Item 7	When encountering difficult situations, I become so stressed that I cannot think of a way to resolve the situation.		0.76
Item 17	I feel I have no power to change things in difficult situations.		0.71
Item 4	When I encounter difficult situations, I feel like I am losing control.		0.69
**Item 2**	**I have a hard time making decisions when faced with difficult situations.**		**0.65**
Item 9	I find it troublesome that there are so many different ways to deal with difficult situations.		0.56
**Item 15**	**I am capable of overcoming the difficulties in life that I face.**		**0.54**
**Item 1**	**I am good at “sizing up” situations.**		**0.41**

*According to the original CFI items in boldface are 13 items of Alternatives subscale and other 7 items are of Control subscale. Extraction method for the exploratory factor analysis is principal axis factoring. Rotation Method is Promax with Kaiser Normalization.*

Based on the results from the EFA two subscales of the CFI-R were constructed. The Alternatives subscale with the eigenvalue of 5.10 explained 25.50% of the total variance, and the Control subscale with the eigenvalue of 2.33 explained 11.64% of the total variance. Internal consistency estimates of reliability, as indexed by Cronbach’s alpha, for the Alternatives subscale and the Control subscale were 0.82 and 0.85, respectively. The means of inter-item correlations were 0.30 for the Alternatives subscale and 0.41 for the Control subscale. Cronbach’s alpha for the CFI-R was 0.85 and it had a mean of inter-item correlations of 0.25. This two-factor CFI-R’s model further tested whether it would fit the data well. The results supported the acceptable model fit scaled χ^2^(*N* = 445) = 428.23, *df* = 169, *p* < 0.001, CFI = 0.88, GFI = 0.89, SRMR = 0.07, RMSEA = 0.06, 90% CI [0.05, 0.07]. The two factors were correlated 0.36 (*p* < 0.001) in the model. For comparison purposes, we examined the original CFI’s factorial structure. We ran CFA using the CFI’s developers scoring procedure (Dennis and Vander Wal, 2010) with the same sample. Results did not indicate a good fit scaled χ^2^(*N* = 445) = 956.97, *df* = 169, *p* < 0.001, CFI = 0.65, GFI = 0.77, SRMR = 0.14, RMSEA = 0.10, 90% CI, [0.10, 0.11]. All fit indices showed a bad fit for a two-factor model of the original CFI, although the two factors were correlated 0.51 (*p* < 0.001) in the model. Notwithstanding the height of the correlations, we observed that a two-factor model of the CFI-R with a specified number of items fitted the data better than a two-factor model of the original CFI. Thus, these analyses confirmed the CFI-R’s factor structure and provided further support for the modification of the original CFI.

### Test–Retest Reliability

The test–retest reliability assessment was conducted with 262 undergraduate students who participated in a general questionnaire session (testing), ranging in age from 16 to 23 years (*M* = 18.40, *SD* = 1.01), 231 females (aged from 16 to 23 years, *M* = 18.40, *SD* = 1.00) and 31 males (aged from 17 to 22 years, *M* = 18.42, *SD* = 1.09). They were invited to return for the re-testing questionnaire session 7-week later. Mean scores on the CFI-R were 106.36 (*SD* = 12.11) at testing and 104.86 (*SD* = 12.20) at re-testing. The mean scores for the Alternatives subscale were 67.02 (*SD* = 7.59) at testing and 66.72 (*SD* = 7.39). The mean scores for the Control subscale were 39.34 (*SD* = 7.45) at testing and 38.15 (*SD* = 7.58). The test–retest correlation had a moderate to strong range for the CFI-R (*r* = 0.68, *p* < 0.01; ICC = 0.68, *p* < 0.001), Alternatives subscale (*r* = 0.67, *p* < 0.01; ICC = 0.67, *p* < 0.001) and Control subscale (*r* = 0.64, *p* < 0.01; ICC = 0.63, *p* < 0.001).

### Construct Validity

To evaluate the construct validity of the CFI-R, we studied associations with other measures of depressive symptoms, coping and rigidity (see **Table 2**). The assessment was conducted with 269 undergraduate students who completed all measures comprising the BDI, ASQ, WC-R, and TRQ. They ranged in age from 17 to 24 years (*M* = 18.57, *SD* = 1.06), 219 females (aged from 17 to 23 years, *M* = 18.46, *SD* = 0.83) and 50 males (aged from 17 to 24 years, *M* = 19.08, *SD* = 1.68). Given that greater cognitive rigidity on the CFI was associated with increasing depressive symptomatology (Dennis and Vander Wal, 2010), the CFI-R was negatively correlated with the BDI. These correlations provided support for the concurrent validity of the CFI-R and its two subscales. Evidence for convergent construct validity of the CFI-R was obtained via examining its correlations with the ASQ. Consistent with the findings of the CFI’s developers (Dennis and Vander Wal, 2010), the ASQ demonstrated significant correlations with the CFI-R total scores and its Control subscale, and a non-significant correlation with the Alternatives subscale. As predicted, CF measured by the CFI was positively associated with the adaptive forms of coping and inversely associated with maladaptive forms of coping. Given that we examined convergent construct validity between the CFI-R and various subscales of the WC-R. The CFI-R total scores showed significant correlations with the Self-controlling, Planful Problem-Solving and Positive Reappraisal coping subscales considered to be adaptive, and significant inverse correlations with the Escape-Avoidance considered to be maladaptive. The correlations between the CFI-R total scores and Confrontive Coping, Distancing, Seeking Social Support, Accepting Responsibility, were not significant. Correlations with the coping subscales were the same for the two CFI-R’s subscales, except the significant correlations of the Control subscale with the Distancing and Accepting Responsibility coping subscales. Support for the convergent construct validity of the CFI-R was obtained via its significant inverse correlations with TRQ. All types of mental rigidity were associated significantly with the CFI-R and its subscales, except the non-significant correlations of two TRQ’s subscales – Rigidity Symptom Complex and Premorbid Rigidity – with the CFI-R’s Alternatives subscale.

**Table 2:** Pearson’s correlation coefficient of the CFI-R with other related measures’ scales.

Measures		CFI-R		
		Total	Alternatives	Control
CFI-R	Total			
	Alternatives	0.823**		
	Control	0.805**	0.327**	
BDI	Total	-0.378**	-0.166**	-0.456**
	Cognitive-affective	-0.379**	-0.154*	-0.470**
	Somatic-performance	-0.306**	-0.156*	-0.348**
ASQ		0.154*	0.082	0.170**
WC-R	Confrontive coping	-0.007	-0.001	0.002
	Distancing	-0.103	-0.033	-0.137*
	Self-controlling	0.267**	0.263**	0.170**
	Seeking social support	-0.003	0.078	-0.087
	Accepting responsibility	-0.117	0.111	-0.311**
	Escape-avoidance	-0.314**	-0.140*	-0.377**
	Planful problem-solving	0.564**	0.485**	0.433**
	Positive reappraisal	0.352**	0.307**	0.266**
TRQ	Rigidity symptom complex	-0.323**	-0.102	-0.432**
	Actual rigidity	-0.476**	-0.249**	-0.532**
	Sensitive rigidity	-0.419**	-0.173**	-0.518**
	Attitudinal rigidity	-0.199**	-0.156*	-0.168**
	Rigidity as a state	-0.493**	-0.218**	-0.594**
	Premorbid rigidity	-0.267**	-0.109	-0.331**

*^∗^*p* < 0.05; ^∗∗^*p* < 0.01. CFI-R, Russian version of Cognitive Flexibility Inventory; BDI, Beck Depression Inventory; ASQ, Attributional Style Questionnaire; WC-R, Ways of Coping (Revised); TRQ, Tomsk Rigidity Questionnaire.*

Given that the CFI was developed to measure the type of CF needed to successfully challenge and replace maladaptive thoughts with more rational and balanced thinking in treatment context (Dennis and Vander Wal, 2010), we examined results for non-clinical and clinical samples to assess whether the construct validity were affected by psychopathology using “known-groups” method. The non-clinical sample (*n* = 35) was randomly drawn from the total sample of undergraduate students. None of them met the DSM-5 depressive, anxiety and personality disorders’ criteria. Levene’s tests of homogeneity of variances revealed non-significant variance equalities among the groups, *F* = 1.148 (*p* > 0.05) for the Alternatives subscale, *F* = 3.205 (*p* > 0.05) the Control subscale, and *F* = 0.270 (*p* > 0.05) for total CFI-R. The *t*-statistic confirmed that the CFI-R’s total score and its subscales’ scores were significantly better for the non-clinical sample than for the clinical sample (see **Table 3**).

**Table 3:** Comparison statistics for the non-clinical and clinical samples.

CFI-R	Non-clinical sample (*n* = 35)		Clinical sample (*n* = 35)		CI_95%_	*t*_(68)_	*p*
	*M*	*SD*	*M*	*SD*			
Total	110.57	11.84	93.34	13.33	[11.21 23.24]	5.72	<0.001
Alternatives	68.34	6.77	61.89	8.63	[2.76 10.16]	3.48	<0.001
Control	42.23	6.81	31.46	8.91	[6.99 14.56]	5.68	<0.001

## Discussion

In the present study we examined the replicability of a two-factor structure and the psychometric properties of the CFI in Russian-speaking university students. Using exploratory and CFA we modified the original CFI. Given that the CFI-R was recommended with appropriate adaptation and further psychometric validation. EFA results indicated good internal consistency reliability for CFI-R and its subscales, and CFA results showed that a two-factor model of the CFI-R with a specified number of items fitted the data better than a two-factor model of the original CFI. The CFI-R was composed of 20 items – 12 items on the Alternatives subscale and 8 items on the Control subscale. Some modifications were made to the CFI to obtain the CFI-R included the revising items 14 and 19, originally developed to assess two aspects of cognitive flexibility: the ability to perceive multiple alternative explanations for life occurrences and human behavior and the ability to generate multiple alternative solutions to difficult situations, and items 1, 2 and 15, originally developed to assess one aspect of cognitive flexibility: the tendency to perceive difficult situations as controllable. It is important to note that Shareh et al. (2014) identified the same set of items as problematic and provided a three-factor solution for the Iranian version of the CFI.

A newly specified structure fitted the data better than the original CFI. All fit statistics indicated acceptable model fit for the model of the CFI-R. Results also provided evidence that the CFI-R and its two subscales had acceptable 7-week test–retest reliability.

The concurrent validity of the CFI-R was confirmed by correlations between scores on the CFI-R and those on other related measures. The CFI-R was significantly and negatively related with BDI indicating that a person with a lower CF on the CFI-R was associated with increasing depressive symptoms on the BDI. Our results revealed a similar finding for associations of the original CFI and its two subscales with depressive symptomology (Dennis and Vander Wal, 2010). We also expected that the CF on the CFI-R negatively associated with rigidity on the TRQ. The expected associations with the CFI-R total score, the Alternatives subscale and Control subscale scores were found for all subscales of the TRQ, except for Rigidity Symptom Complex subscale and Premorbid Rigidity subscale. For those subscales the associations with the CFI-R’s Alternatives subscale were non-significant. This indicates that the TRQ’s Rigidity Symptom Complex subscale and Premorbid Rigidity subscale measure weakly related concepts. For example, the Premorbid rigidity subscale refers to the consideration whether a person has experienced difficulties in the situations regarding any chances being in teen-age and preadult age (e.g., “I have preferred to follow my own habits and tastes since childhood” or “In childhood and adolescence, I often invented something new and did things over”). As for the Rigidity Symptom Complex subscale, it measures a unique construct not measured by those of the Alternatives subscale [aspects of cognitive flexibility: (b) the ability to perceive multiple alternative explanations for life occurrences and human behavior, and (c) the ability to generate multiple alternative solutions to difficult situations].

Furthermore, research has also shown correlations between the CFI-R and the ASQ. Greater cognitive rigidity on the ASQ was associated with greater CF on total CFI-R and the Control subscale, except with the Alternatives subscale. Although the results were consistent with the findings of the CFI developers, in this research we observed the significant associations between total CFI-R and both subscales and the rigidity subscales on the TRQ. Hence, further study is needed to better understand whether the construct of cognitive rigidity as measured by Teasdale et al. (2001) is in line with the theoretical prediction for aspects of CF as measured by Dennis and Vander Wal (2010).

There was also evidence of convergent construct validity of the CFI-R. As expected based on prior research (Dennis and Vander Wal, 2010), the scores indicative of greater CF on total CFI-R and both subscales were associated with an increased tendency to utilize coping strategies considered to be adaptive – Self-controlling, Planful Problem-Solving and Positive Reappraisal Coping – and the decreased tendency to utilize coping strategies considered to be maladaptive – Escape-Avoidance. Despite having associations with the WC-R, the CFI-R’ the Alternatives and Control subscales had different relationships with coping subscales. Greater CF on the Control subscale but not the Alternatives subscale was significantly associated with a decreased tendency to utilize Distancing and Accepting Responsibility as coping strategies. This suggests that individuals who perceive difficult situations as controllable perform adaptive coping rather than rigid thinking styles associated with efforts to detach themselves and acknowledge their own role in the problem. Our finding is in line with Dennis and Vander Wal’s theoretical prediction for the two-factor structure of the CFI, which measures aspects of CF that differentially affect an individual’s reaction to experiencing challenging life events.

Additionally preliminary support for the construct validity of the CFI-R was obtained via examining the known-groups validity. The results indicate that the CFI-R scores for participants who met the DSM-5 depressive, anxiety and personality disorders’ criteria were significantly worse than for participants who did not. This finding suggests that individuals with depressive symptoms display lower CF on the CFI-R which is indicative of greater cognitive rigidity associated with less cognitive adaptability when encountering stressful situations (Dennis and Vander Wal, 2010).

Overall, the results of this study presented acceptable fit of the two-factor structure of the Russian language version of the CFI among a sample of university student, suitable reliability and validity with the exception of the associations between the CFI-R and AQS, which are unclear. This study suggests that psychometric properties of the Russian CFI are comparable to the English original, making it appropriate to research assessment of the type of CF necessary to successfully challenge and restructure maladaptive beliefs with more balanced and adaptive thinking in Russian speaking population.

## Limitation and Future Research Directions

Several limitations should be considered in interpreting the study results. Firstly, we started from the Russian translation of the CFI and then confirmed the reliability and validity of its two-factor structure (with a specified number of items) that was consistent with the English original. This suggests that some impact of cross-cultural factors might play a role in the results of the factor analysis. Secondly, given that participants across the samples were predominantly female, a special focus should be made on the generalization of the results. It would be necessary to examine the influence of gender on the CFI-R total score and both subscales’ scores. Thirdly, the participants comprised the non-clinical sample. Future studies would benefit from assessing the reliability and validity of the CFI-R using clinical samples. A larger sample of clinical individuals would allow for a direct comparison of the factor structure in non-clinical and clinical samples. However, it is difficult to find individuals that are very similar in terms of their demographic and clinical characteristics.

## Conclusion

The findings of the study provide evidence for the satisfactory psychometric properties of the CFI by applying it to a sample of Russian university students, a population not previously studied. The factor structure of the CFI-R with a specified number of items is consistent with a two-factor model and showed an acceptable model fit. The internal consistency and test–retest reliability for the CFI-R and its two subscales were also adequate. As predicted, we indicated the negative associations of CF aspects measured by CFI-R with depressive symptomology, coping strategies considered to be maladaptive, and personality rigidity. The study succeeded in increasing the applicability of the Russian CF Inventory in research context and facilitated its utility as a measure in the treatment context.

## Author Contributions

SK and EO conceived and designed the study and analyzed the data. EO carried out the implementation of the research. SK took the lead in writing the manuscript. All authors discussed the results and contributed to the final manuscript.

## Conflict of Interest Statement

The authors declare that the research was conducted in the absence of any commercial or financial relationships that could be construed as a potential conflict of interest.

## Footnotes

1 It is a set of L scale’s items extracted from the Eysenck Personality Inventory (Eysenck and Eysenck, 1964).
